# Lung Diseases: Chronic Respiratory Infections

**DOI:** 10.3390/ijms19103051

**Published:** 2018-10-07

**Authors:** Francesco Blasi

**Affiliations:** 1 Department of Pathophysiology and Transplantation, Università degli Studi di Milano, 20122 Milan, Italy; francesco.blasi@unimi.it; Tel.: +39-025-032-0627; Fax: +39-025-032-0625; 2 Internal Medicine Department, Respiratory Unit and Adult Cystic Fibrosis Center, Fondazione IRCCS Cà Granda Ospedale Maggiore Policlinico Milan, 20122 Milan, Italy

Acute and chronic respiratory infections are the leading causes of morbidity and mortality worldwide [1]. A better understanding of the epidemiology, pathophysiologic mechanisms and potential new treatments of chronic respiratory infections is one of the main issues in the management of chronic respiratory infections.

In this special issue, 9 original research studies and 5 reviews have been published (see Table 1).

The first group of articles analyzes different possible pathways of the immune and inflammatory response, before proposing possible diagnostic and treatment interventions [2,3,4,5].

Douglas et al. [2] analyzed the evidence from the literature on the enhancement of upper respiratory innate immunity due to bitter taste receptors and the possible roles of individual taste differences in the clinical management of patients with upper respiratory infections. The main bitter taste receptor, T2R38, responds to bitter compounds produced by invading bacteria, which potentiates the immunological response through the innate response. The authors suggest that the possible role of bitter taste receptors could be a target for therapeutic interventions aimed to enhance the immune response to bacteria.

The potential role of matricellular proteins as immunomodulators is addressed in the paper by Shiratori et al., which analyzed the plasma levels in Japanese patients affected by pulmonary tuberculosis or latent tuberculosis compared to healthy controls [3]. The correlations between matricellular proteins, such as osteopontin, soluble CD44 and galectin-9, and severity scores seems to indicate that these proteins can be predictors of tuberculosis-related inflammation and clinical severity.

The role of anti-inflammatory compounds in preventing lung injury was assessed in the original research by An et al. [4]. In an animal model, using lipopolysaccharide (LPS) tracheal instillation, the authors identified Polydexyribonucleotide (PDRN) as a potent agent for reducing the excessive apoptosis that plays a key role in the progression of lung injury induced by LPS, suggesting that PDRN should be evaluated as a potential therapeutic agent for the treatment of lung injuries.

The regulation of inflammatory processes in the lung through the new potential targets was analyzed in the original research published by Florence et al. [5]. The authors demonstrate that Bruton’s tyrosine kinase (Btk) and matrix metalloproteinase-9 (MMP-9) specific siRNA can down-regulate lung inflammation in a mice model. Both Btk and specific inhibitors of MMP-9 are suggested as potential therapeutic targets.

The second group of papers addresses the control of difficult-to-treat Gram-negative bacteria that are associated with recurrent and/or persistent lung infections [6,7,8,9].

Chronic *Pseudomonas aeruginosa* infections are associated with high inflammation levels in the airways and in the lung. Heparan sulfate competitors have been evaluated by Lorè et al. as possible anti-inflammatory compounds [6]. The authors analyzed the efficacy of different heparan sulfate competitors in reducing leukocyte recruitment, cytokine/chemokine production and bacterial burden that is associated with acute and chronic *Pseudomonas* infections using both in vitro and in vivo models.

N-acetyl heparin and a glycol-split heparin resulted in decreased inflammation, biofilm formation and bacterial burden, suggesting that these compounds can be novel therapeutic approaches for *Pseudomonas* infections.

*Burkholderia cepacia* complex (BCC) is a difficult-to-treat group of opportunistic pathogens that mainly affect cystic fibrosis and immunocompromised patients. Carnell et al. [7] analyzed the potential antimicrobial efficacy and effect of a new antimicrobial compound S-(4-chlorobenzyl)isothiourea hydrochloride (Q22) on the virulence-related traits of BCC bacteria. This drug is an inhibitor of one cytoskeletal protein, which is namely the actin homolog MreB.

Unfortunately, Q22 appears to enhance the BCC virulence and proinflammatory potential in an *in vitro* model. Moreover, in the *in vivo* model, exposure to Q22 seems to increase the level of resistance to H_2_O_2_-induced oxidative stress by BCC strains and the compound was toxic to the mice.

Bragonzi et al. [8] reported the ability of a BCC Mex1 strain to rapidly establish respiratory tract chronic infections in mice following serial passages. This capacity is apparently not related to phenotypic and genetic changes, but is probably linked to an increased virulence.

Microbiome gene repertoire in the airways of cystic fibrosis patients with severe lung disease has been evaluated by Bacci et al. [9]. Metagenomics investigation of the bacterial communities resulted in the identification of a high prevalence of genes that have been related to antibiotic resistance and virulence mechanisms in patients with more severe disease.

The third group of articles analyzed fungi and non-tuberculous mycobacteria (NTM) epidemiology and potential new treatment approaches in patients with bronchiectasis and cystic fibrosis [10,11,12]. Everaerts et al. reported the results of a study addressing the potential role of galactomannan detection in the induced sputum of COPD and COPD–bronchiectasis overlap patients for the diagnosis of *Aspergillus fumigatus* infections [10]. Patients with COPD–bronchiectasis overlap have a higher rate of positive results. The authors suggest that galactomannan detection in induced sputum may provide a sensitive marker for *Aspergillus fumigatus* infections.

In the same line, Maiz et al., in a concise review, analyzed the role of fungal infections in patients with bronchiectasis [11]. The authors discussed the problems related to the diagnosis, epidemiology and clinical significance. Moreover, the need for further research into the lung mycobiome and its interactions with viral and bacterial microbiota in the pathogenesis of bronchiectasis was underlined.

In the last few years, an increasing interest in NTM pulmonary involvement has been reported in different diseases [16]. Faverio et al. reported an observational, prospective study describing the management, in real life, of NTM pulmonary infections in a cohort of 261 adult bronchiectasis patients [12]. In 12% of these patients, a NTM pulmonary infection has been demonstrated with an association with cylindrical bronchiectasis, a history of weight loss and a “tree-in-bud” radiological pattern. Only 1/3 of these patients achieved culture conversion without recurrence. This study shows a fairly high incidence of NTM infection and gives some insights on the possible clinical parameters that are associated with an increased risk of NTM infection.

Inhaled antibiotic therapy in chronic respiratory diseases is another important topic analyzed in this special issue [13,14]. Inhaled antibiotic therapy has many potential benefits in the management of chronic respiratory infections, which are mainly related to the high concentration in the target site, increasing the potential efficacy and reducing systemic exposure by minimizing the toxicity [17]. Maselli et al. reviewed the potential role of inhaled antibiotic treatment in patients with cystic fibrosis, bronchiectasis and NTM pulmonary infections [13]. In cystic fibrosis, inhaled antibiotics have been demonstrated to significantly improve the disease management by reducing exacerbations in addition to improving lung function and quality of life [18].

Inhaled antibiotic treatment efficacy in bronchiectasis is still an open and challenging question. No inhaled antibiotics have been approved in this indication even if the experts indicate that this therapy is a treatment of choice for the management of chronic respiratory infections in these patients [19].

Maselli et al. also analyzed the data on the use of this approach in NTM pulmonary infections, reporting promising results of inhaled liposomal amikacin, which was recently confirmed by the FDA approval of one formulation for human use [20].

COPD is another respiratory disease where antibiotics are largely used. Miravitlles et al. reviewed the role of antibiotics in treating and preventing COPD exacerbations [14]. Antibiotics should be reserved for the treatment of exacerbations of patients with severe disease and presenting a cluster of symptoms, including increased sputum purulence and worsening dyspnea. Long-term preventive therapy with antibiotics is controversial and should be used cautiously due to the potential side effects, increase in resistance rate and microbiome alterations.

The microbiome is increasingly reported as a potential actor in the pathogenesis of idiopathic pulmonary fibrosis [21]. In this special issue, Fastres et al. analyzed the potential role of the lung microbiome as a therapeutic target in idiopathic pulmonary fibrosis [15]. The authors conclude that antibiotic therapy, particularly long-term, may have a role in controlling exacerbations and immunomodulating the inflammatory response.

In conclusion, I would like to thank all the authors who contributed to this Special Issue. The articles that were published illustrate the advances in the research in chronic respiratory infections, which provides important insights that will help all the clinicians in improving the diagnosis and management of these important diseases.

## Figures and Tables

**Table 1:** Contributions to the special issue "Lung Diseases: Chronic Respiratory Infections".

Authors	Title	Type	Key Messages
Douglas JE et al. [2]	Taste Receptors Mediate Sinonasal Immunity and Respiratory Disease	Review	Upper airway epithelium bitter taste receptors stimulation, specifically T2R38, potentiate the local innate immune response
Shiratori B et al. [3]	Immunological Roles of Elevated Plasma Levels of Matricellular Proteins in Japanese Patients with Pulmonary Tuberculosis	Original Research	Matricellular proteins, including osteopontin and galectin-9, seems to have an immunoregulatory, rather than inflammatory, effect in the context of TB pathology
An J et al. [4]	Polydeoxyribonucleotide ameliorates lipopolysaccharide-induced lung injury by inhibiting apoptotic cell death in rats	Original Research	In an animal model, polydexyribonucleotide (PDRN) demonstrated an anti-inflammatory effect, decreasing inflammatory cytokines, and suppressing apoptosis. Further studies will address the possible use of PDRN as a new treatment of lung injury.
Florence JM et al. [5]	Disrupting the Btk pathway suppresses COPD-like lung alterations in atherosclerosis prone ApoE^−/−^ mice following regular exposure to cigarette smoking	Original Research	Bruton’s tyrosine kinase (Btk) is involved in the regulation of inflammatory processes in the lungs by regulating the expression of matrix metalloproteinase-9 in the alveolar compartment. In an animal model, the pharmacological inhibition of Btk showed protective effects in the lung exposed to cigarette smoke
Lorè NI et al. [6]	Synthesized heparan sulfate competitors attenuate *Pseudomonas aeruginosa* lung infection	Original research	Competitors of heparan sulfate, N-acetyl heparin and glycol-split heparin reduce leukocyte recruitment and cytokine/chemokine production in an animal model of acute and chronic *P. aeruginosa* pneumonia. In vitro data suggest a reduction in biofilm formation
Carnell SC et al. [7]	Targeting the bacterial cytoskeleton of the *Burkholderia cepacia* complex for antimicrobial development: a cautionary tale	Original Research	Bacterial cytoskeleton destabilizing compounds seem to be potentially harmful in the treatment of *Burkholderia cepacia* complexes as it induces an increase in bacterial virulence factors.
Bragonzi A et al. [8]	Enviromental *Burkholderia cenocepacia* strain enhances fitness by serial passages during long-term chronic airway infections in mice	Original research	Multiple passages of *Burkholderia cenocepacia* are associated with an increased ability to induce chronic lung infections in an animal model with clones with high virulence
Bacci G et al. [9]	A different microbiome gene repertoire in the airways of cystic fibrosis patients with severe lung disease	Original Research	Analysis of the microbiome in severe lung disease of cystic fibrosis patients has shown that there is an increase in virulence- and resistance-related genes.
Everaerts S et al. [10]	*Aspergillus fumigatus* detection and risk factors in patients with COPD–bronchiectasis overlap	Original Research	*Aspergillus fumigatus* presence in the airways is prevalent in COPD patients with bronchiectasis, particularly in the presence of steroid treatment
Maiz L et al. [11]	Fungi in bronchiectasis: a concise review	Review	*Candida albicans* and *Aspergillus fumigatus* appear to be the most prevalent fungi isolated in bronchiectasis
Faverio P et al. [12]	Characterizing non-tuberculous Mycobacteria infections in bronchiectasis	Original Research	In a prospective, observational study of 261 adult bronchiectasis patients, non-tuberculous mycobacteria (NTM) infections have been evaluated. NTM isolation seems to be a frequent event in bronchiectasis patients. Cylindrical bronchiectasis, a CT “tree-in-bud” pattern and a history of weight loss are parameters that might help to suspect the occurrence of a NTM infection.
Maselli DJ et al. [13]	Inhaled antibiotic therapy in chronic respiratory disease	Review	The review analyzes the evidence on the use of inhaled antibiotics in patients with cystic fibrosis, bronchiectasis and non-tuberculous mycobacteria (NTM) infections. Further studies are needed to define the role of inhaled antibiotics.
Miravittles M et al. [14]	Chronic respiratory infections in patient with chronic obstructive pulmonary disease: what is the role of antibiotics?	Review	Chronic infection is associated with COPD exacerbations. Antibiotic use in acute events is controversial but may be important in patients with higher risk of poor outcomes. Antibiotic prophylaxis remains controversial
Fastrès A et al. [15]	The lung microbiome in idiopathic pulmonary fibrosis: a promising approach for targeted therapies	Review	The literature analysis seems to indicate the need for clinical trials of long-term antibiotherapy to see if can act as an immunomodulator and an antibioprophylaxis to prevent acute exacerbations
